# Effects of Selected Socio-Demographic Characteristics of Community Health Workers on Performance of Home Visits during Pregnancy: A Cross-Sectional Study in Busia District, Kenya

**DOI:** 10.5539/gjhs.v4n5p78

**Published:** 2012-07-26

**Authors:** Ndedda Crispin, Annah Wamae, Meshack Ndirangu, David Wamalwa, Gilbert Wangalwa, Patrick Watako, Elijah Mbiti

**Affiliations:** 1 Division of Child and Adolescent Health, MOPHS, Kenya; 2 Department of Family Health, MOPHS, Kenya; 3 AMREF, Kenya Country Office; 4 Busia Child Survival Project (BCSP); 5 Busia Child Survival Project; 6 District Health Management Team Member, Busia District, Kenya; 7 Micronutrient Initiative, Kenya

**Keywords:** socio-demographic characteristics, performance, home visits, pregnancy, community health workers

## Abstract

**Objective:**

Appropriate performance of home visits facilitates adoption of best practices at home and increased demand for facility based services.

**Methods:**

It was a cross-sectional study in which community health workers were observed conducting home visits during pregnancy. Data was collected using a structured questionnaire and the Consultant Quality Index (CQI-2 tool) on record keeping, use of job aids, counselling, client satisfaction and client enablement. Descriptive and inferential statistics were used. Relationships were determined using chi square and odds ratios.

**Results:**

The study showed significant relationships of age with good record keeping (p = 0.0001), appropriate use of job aids (p=0.0001), client satisfaction (p = 0.018) and client enablement (p = 0.001). Male CHWs were 1.6 times more likely to keep better records than females (OR 1.64 CI (1.02-2.63), while females were more likely to counsel and enable their clients OR 0.42 CI (0.25-0.71) and OR 0.29 CI (012-070) respectively when compared to men. Moreover, higher levels of education were associated with good record keeping OR 0.30 CI (0.19-0.49), p=0.0001; appropriate use of job aids OR 0.30 CI (0.15-0.61) and to appropriately counsel their clients OR 0.34 CI (0.20-0.58) than their lower literacy level counterparts. Experience of CHWs was associated with appropriate use of job aids (p = 0.049); client satisfaction (p = 0.0001) and client enablement (p = 0.032).

**Conclusions:**

Socio-demographic characteristics of community health workers affect the performance of home visits in various ways. The study also confirmed that CHWs with lower literacy levels satisfy and enable their clients effectively.

## 1. Introduction

A community health Worker (CHW) is any health worker carrying out functions related to health care delivery; trained in some way in the context of the intervention and having no formal professional or paraprofessional certificate, degree or tertiary education (Lewin et al., 2005). Community Health Worker are considered as a third health service delivery work-force (Sein, 2006). The CHWs have evolved with community based healthcare programmes. However, the titles, the profile and the deployment of CHWs have varied enormously across countries, conditioned by their aspirations and economic capacity (Lehmann & Sanders, 2007).

Evaluation of community health workers’ performance in general, is the focus of much attention at this time, as many countries invest in them as a strategy for the achievement of the millennium development goals (Haines et al., 2007). The effectiveness of Community Health workers (CHWs) has been demonstrated in some studies for example, a CHW programme in India resulted in significant reduction of low birth weight, preterm births and neonatal sepsis (Bang, Baitule, Reddy, & Deshmukh, 2005). In Cambodia, CHWs were effective in community management of malaria (Yeung, Van Damme, Socheat, White, & Mills, 2008) and in identification of pneumonia in Uganda (Kallander et al., 2006). CHW’s effectiveness is enhanced by the fact that they are local residents, and in principle, always accessible to the villagers. Large centrally managed CHWs’ programmes have failed whilst true community based ones work well (Friedman, 2005).

Inadequate performance by CHWs is a widespread problem in many public health fields as demonstrated in malaria control (Morrow, 2009). Studies have also differed on whether socio-demographic factors are important determinants of CHWs’ effectiveness (Lehmann & Sanders 2007). Understanding how the socio-demographic factors influence CHWs’ effectiveness in conducting home visits is therefore of paramount importance primarily for the adoption of evidence based maternal, newborn, child health and nutrition best practices and to increase demand for facility based services including skilled birth attendance.

The roles of CHWs include among others: home visits, environmental sanitation, provision of water supply, first aid, treatment of minor and common illness, nutrition counselling, health education and promotion, surveillance, maternal health, family planning, child health, communicable disease control, community development, referrals, record keeping and data collection (Lehmann & Sanders, 2007).

Community health workers’ programmes usually face a myriad of challenges including selection (Jobert, 1985; Ruebush, Weller, & Klein, 1994), low level or no education, a lack of professional training in health (Brown et al., 2006), the nature of services and workload (Lehmann & Sanders 2007), inter-relationships between the CHWs, facility health workers and community members (Ballester, 2005) and unclear remuneration/motivation mechanisms (Ballester, 2005) amongst many others. Additionally, the management and supervision of these important staff is challenging for example whether they are accountable to the communities, the health care system or non-Governmental organizations. Their supervisors, ironically, may not have supervisory skills thus compounding the problem. Generally, both men and women are recruited as CHWs at grass-root level although females dominate. Most programmes consist of mature and married CHWs (Lehmann & Sanders, 2007).

Studies over time have shown that older CHWs are more respected in their communities (Bhattacharyya, Winch, LeBan, & Tien, 2001). Among some communities such as the Somali, male CHWs find it difficult to pass messages to women (Bentley, 1989). In other communities, resistance from husbands was identified as a key barrier to the participation of women (Brown, Malca, Zumaran, & Miranda, 2006).

Many but not all CHW programmes require literacy as a pre-requisite (Kaseje, Sempebwa & Spencer, 1987; Bentley, 1989; Delacollette, Stuyft, & Molima, 1996; Brown et al., 2006). For example, Kenyan AMREF programmes require seven years of primary education (Chagula & Tarimo, 1975; Johnson & Khanna, 2004) while a community self-help health development programme in Sarididi, Kenya did not consider literacy as selection criteria (Kaseje et al., 1987). Some programmes consider ability to read and write and communication skills (Ande, Oladepo, & Brieger, 2004). Literate CHWs also tend to be younger (Bhattacharyya et al., 2001). However, studies have shown that on one hand, CHWs with higher educational qualifications have opportunities for alternative employment and therefore migrate from one job to another (Brown et al., 2006). On the other hand those with higher education could learn and enhance their skill in the diagnosis of common illness (Bentley, 1989; Kelly et al., 2001; Ande et al., 2004) and thereby deliver better care to the community. Whilst this might be true in some cases, a study in Uganda, found that on the contrary factors like age, sex, education and number of offspring have no effect on CHWs ability to classify Pneumonia and provide treatment accordingly (Kallander et al., 2006).

Few if any studies have investigated the quality of home visits performed by community health workers. This study investigated the effect of selected socio-demographic characteristics on the performance of home visits by CHWs. It is envisioned that findings of this study would inform policy to better implement the community strategy for optimal results.

## 2. Methods

### 2.1 Study Area

The study was undertaken in Funyula and Butula divisions of Busia District, Kenya. Busia District is south of the equator in Western Province and borders Uganda to the West. This is the area where the Division of Child and Adolescent Health conducted a pilot study on community based maternal and newborn care through the community strategy in collaboration with the African Medical and Research Foundation (AMREF).

### 2.2 Sampling

The study area had 700 community health workers involved in the implementation of the project spread over seven supervisory areas. Five of these supervisory areas were selected based on CHW population proportion to size. Using lists of CHWs in these areas, 19 were randomly selected from each supervisory area for inclusion in the study. The selected supervisory areas were: Supervisory area 1-Butula Bujumba Bumala; supervisory area 2-Butula Marachi Central; supervisory area 3-Butula Lugulu Elukhari; supervisory area 4-Samia Nambuku Namboboto and supervisory area 5-Samia Nanguba Bujwang’a. Using a cross-sectional design, the study explored the performance of home visits during pregnancy by community health workers involved in community based maternal and newborn care over the study period.

### 2.3 Data Collection and Tools

A supervisory checklist adapted from one used in India (Bang et al., 2005) was used to document home visit observation while the consultant quality instrument (CQI) developed by Howie et al. (2000) (www.biomedexperts/CQI-2) for client exit interviews was used to determine client satisfaction i.e. the client’s perception of the home visit and enablement (the ability of the consultation to result in client behaviour change). Each of the selected community health workers was observed conducting five home visits to pregnant women and the performance of health communication during the consultation was then documented on a structured checklist and an exit interview conducted thereafter by a separate researcher. 

### 2.4 Variables

The independent variables were age divided into under 30 years, 30 to 40 years, 40 to 50 years 50 to 60 years and above 60 years sex (male and female) marital status divided into single, married, widow/widower; highest level of education was divided into primary, secondary and above; experience was segmented into 1-2 years, 3-5 years, 6-10 years and above 10 years. The dependent variables were scored on scale record keeping (poor and good), use of job aids (appropriate and inappropriate), counselling skills (appropriate and inappropriate), client satisfaction (low client satisfaction and high client satisfaction) and finally client enablement divided into (client enabled and client not enabled).

### 2.5 Data Analysis

Data was cleaned and entered into SPSS version 18 software for analysis. Descriptive statistics were computed and relationships and significant tests determined using Chi square and Odd Ratios (ORs). The OR is used to assess the possibility of a particular outcome, (performance) if a certain exposure (socio-demographic variable), is present. Further analysis was done using LQAS to compare performance by supervisory area.

## 3. Results

A total of 378 community health worker home visit consultations were observed. Each CHW conducted five home visits staggered over the study period. The CHWs were predominantly female 60% male 40%. Their ages ranged from 23 to above 60 years. The majority were between 31 and 40 years (59.3%) followed by the age group 40 to 50 years (21.2%). The under 30 year old made up 15.3%. Only 4.2% of the CHWs were above 50 years. Most of the community health workers were married (95.8%).

In regard to the level of education, 67.7% of CHWs had completed secondary school, 30.2 % primary level of education 30.2% and (2.1% had attained tertiary education with similar percentage having no formal education at all. Distribution of level of education was equal among male and female CHWs. There were disparities in the level of education in the five supervisory areas. In supervisory area four Samia Nambuku Namboboto 84.2% of the CHWs had secondary and above level of education therefore having the highest proportion of literate CHWs. Supervisory area three had the lowest level 43.7% of secondary-and-above education.

Results showed that 79.9% of the CHWs had worked for 3 to 5 years, 10.6% for 6 to 10 years, 4.2% had worked for more than 10 years as community health workers. Only 5.1% had less than 3 years working experience.

### 3.1 Effect of Age on Performance

Table 1 shows that there are strong relationships between age and good record keeping (p = 0.0001), appropriate use of job aids (p = 0.0001), client satisfaction (p = 0.018) and client enablement (p = 0.001). Table 1 shows that there is no relationship between age and gender (p = 0.129). Chi square computations further showed that community health workers aged 40 to 50 years kept best records and also used job aids most appropriately followed by the age group 30 to 40 both parameters. It was further established that the age group 40 to 50 had the best overall performance of home visits. Those aged 30 to 40 years performed very well in record keeping, use of job aids and client enablement but surprisingly their clients were not satisfied. Those above 60 years kept the worst records, however, this age group enabled their clients more than any other age group. Elderly CHWs were found to have low literacy. It was observed that all age groups had sub-optimal performance in regard to client satisfaction. Client satisfaction appeared to be weakest above 50 years. The young CHWs performed very well in client enablement and also optimally on counselling and appropriate use of job aids. They were a little weak in record keeping and weakest in client satisfaction. There was no relationship of age with appropriate counselling of clients.

**Table 1:** Effects of age on performance

	Under 30 years	30 to 40 years	40 to 50 years	50 to 60 years	Above 60 years	TOTAL	χ^2^
**Records**							
Good record keeping	32	170	64	4	0	270	
Poor records	26	54	16	8	4	108	**P=0.0001**
**Use of Job aids**							
Appropriate use	46	204	80	8	4	342	
Inappropriate use	12	20	0	4	0	36	**P=0.0001**
**Counselling**							
Apprppropriate	48	188	58	8	4	306	
Inappropriate	10	36	22	4	0	72	**P=0.105**
**Client satisfaction**							
Low satisfaction	16	76	34	0	0	126	
High satisfaction	42	148	46	12	4	252	**P=0.018**
**Client enablement**							
Client not enabled	56	214	72	8	4	354	
Client enabled	2	10	8	4	0	24	**P=0.001**

### 3.2 Effect of Sex on Performance

Table 2 shows a significant relation between sex of the CHW and good record keeping (p= 0.042), appropriate counselling (p = 0.001), and client enablement (p = 0.004). Further tests were undertaken by calculating the Odds Ratio. Table 2 shows that male CHWs were 1.6 times more likely to keep better records than females. On the other hand, female CHWs were 58% more likely to counsel their clients appropriately than the males OR 0.42 95% CI (0.25-0.71) while the males were 71% more likelyto enable clients than the females OR 0.29 95% CI (012-070). There was no significant association between gender and appropriate use of job aids and client satisfaction.

**Table 2:** Effects of sex on performance

	Male	Female	Total	χ^2^
**Records**				
Good record keeping	113	157	270	
Poor record keeping	33	75	108	**P=0.042**
OR	1.64(1.02-2.63)	Ref		
**Use of Job aids**				
Appropriate use	130	212	342	
Inappropriate use	16	20	36	**P=0.451**
OR	0.77(0.38-1.53)	Ref		
**Counselling**				
Appropriate	106	200	306	
Inappropriate	40	32	72	**P=0.001**
OR	0.42(0.25-0.71)	Ref		
**Client satisfaction**				
High satisfaction	54	72	126	
Low satisfaction	92	160	252	**P=0.232**
OR	1.30(0.84-2.02)	Ref		
**Client enablement**				
Client enabled	130	224	354	
Client not enabled	16	8	24	**P=0.004**
OR	0.29(012-070)	Ref		

### 3.3 Effects of Level of Education

Table 3 shows that levels of literacy amongst community health workers is associated with good record keeping, use of job aids and counselling but not satisfaction and enablement. Table 3 further shows that community health workers with higher levels of education were 70% more likely to keep good records and use job aids more appropriately than less literate ones. They were also 64% more likely to counsel their clients more appropriately. The results show no association between education level of CHWs and client satisfaction (p = 0.057) and client enablement (p = 0.726).

**Table 3:** Effects of education level on performance of CHWs

	Primary	Secondary and above	Total	χ^2^
**Records**				
Good record keeping	61	209	270	
Poor record keeping	53	55	108	**P=0.0001**
OR	0.30(0.19-0.49)	Ref		
**Job aids**				
Appropriate use	94	248	342	
Inappropriate use	20	16	36	**P=0.0001**
OR	0.30(0.15-0.61)	Ref		
**Counselling**				
Appropriate	78	228	306	
Inappropriate	36	36	72	**P=0.0001**
OR	0.34(0.20-0.58)	Ref		
**Satisfaction**				
High satisfaction	30	96	126	**P=0.057**
Low satisfaction	84	168	252	
OR	0.63(0.38-1.02)	Ref		
**Enablement**				
Client enabled	106	248	354	
Client not enabled	8	16	24	**P=0.726**
OR	0.85(0.36-2.06)	Ref		

### 3.4 Effects of Work Experience

Chi square tests showed a relationship of experience of CHWs with appropriate use of job aids (p = 0.049), client satisfaction (p = 0.0001) and client enablement (p = 0.032) (Table 4). There was no association of experience with record keeping (p=0.398) and counselling of clients (p = 0.929). Furthermore, Community Health Workers who had worked for 3 to 5 years had the best records, used job aids most appropriately, and also counselled clients most appropriately. This group also enabled their clients very well. It can be concluded that although this group had a little weakness in client satisfaction, it had the best overall performance of home visits. Highly experienced CHWs satisfied and enabled their clients more than the less experienced counterparts. It was noted that those who had worked for more than ten years performed all the home visit tasks above average (Table 4).

**Table 4:** Effects of experience on performance

	1 to 2 years	3 to 5 years	6 to 10 years	Over 10 years	Total	χ^2^
**Records**						
Good record keeping	11	219	28	12	270	
Poor record keeping	9	83	12	4	108	**P=0.398**
**Job aids**						
Appropriate use	16	278	36	12	342	
Inappropriate use	4	24	4	4	36	**P=0.049**
**Counselling**						
Appropriate	16	246	32	12	306	
Inappropriate	4	56	8	4	72	**P=0.929**
**Satisfaction**						
High satisfaction	0	106	8	12	126	
Low satisfaction	20	196	32	4	252	**P=0.0001**
**Enablement**						
Client enabled	16	286	36	16	354	
Client not enabled	4	16	4	0	24	**P=0.032**

### 3.5 Other Significant Findings

The participating CHWs were drawn from five distinct supervisory areas. A comparative analysis using LQAS showed that cumulatively, some areas had more CHWs having secondary and above level of education than others. Area 3 Butula Lugulu Elukhari had the lowest proportion of high literacy CHWs while area 4 Samia Nambuku Namboboto had the highest proportion of high literacy CHWs. Comparison of performance of home visits in these areas revealed that areas with low literacy level CHWs could counsel appropriately (decision rule 11, score 13), establish high client satisfaction (decision rule 3, score 4) and enable clients (decision rule 16, score 17) just as well as, if not better than those areas with high literacy level CHWs. This finding is significant given that the ultimate goal of health communication is client enablement which is the ability to convince clients adopt evidence based care practices. Low literacy CHWs can achieve this.

## 4. Discussion

This study aimed at establishing how selected socio-demographic characteristics of community health workers affected the delivery of home visits during pregnancy. Results showed that age, sex, level of education, and experience of the community health workers affected the performance of record keeping, use of job aids, counselling on care during pregnancy, client satisfaction and client enablement. There are however, limited similar studies for comparison.

The mature age of the community health workers is related to appropriate counselling and client enablement. The age-group 30 to 40 appeared to be the most appropriate for selection of community health workers in order to obtain optimum results. Younger and much older CHWs had sub-optimal performance. This differs from a study in Uganda (Kallander et al., 2006) which found that factors such as age, sex and education had no effect on the CHWs’ performance.

Sex of the community health worker was related to good record keeping, counselling and client enablement with female CHWs counselling and enabling their clients better than their male counterparts. This also contrasts with the Uganda study (Kallander et al., 2006) which found no relation of sex with performance. This finding would favour having female community health workers specialize in maternal, newborn, child health and nutrition interventions. The study did not find any relation of marital status with performance.

A higher education level was related to better performance in all parameters except client enablement. This result concurs with a study in Nigeria (Ande, 2004) which observed that literate CHWs could learn and enhance skills and therefore deliver services better. The study however, showed that CHW education level had no influence on enabling clients to adopt best practices. This is an unfortunate scenario given that the ultimate purpose of health communication is behaviour change. A comparison of performance by supervisory areas showed that areas with low literacy CHWs could counsel, satisfy and enable clients effectively therefore agreeing with other studies such as one conducted in Uganda (Kallander et al., 2006). This finding implies that low literacy or illiterate community members should not be discriminated against during selection agreeing with the Sarididi study (Kaseje et al., 1987) in which education was not a selection criterion for CHWs.

Experienced CHWs were found to be most effective at establishing client satisfaction and client enablement both of which are very important for behaviour change and demand creation for services.

## 5. Conclusion

The study established that age, sex, level of education and experience of community health workers are important characteristics to consider in CHW programmes. Community health workers aged 30 to 50 are most appropriate for selection of CHWs partly because this age group is not only still energetic but is apparently socially settled and will likely exhibit lower attrition levels. Female CHWs are best suited to undertake maternal, newborn, child health and nutrition interventions because they counsel and enable clients better than their male counterparts. The fact that areas with low literacy level CHWs could perform home visits effectively by satisfying and enabling their clients implies that CHW programmes can be implemented in all areas particularly in low income countries with limited access to education. This is despite the fact that literate CHWs grasp concepts quicker.

### Authors’ Contributions

NC conceived the study, designed the protocol, analyzed results and drafted the manuscript. AW and MN contributed to the design and in editing the protocol and manuscript DW was the Programme manager of the Busia Child Survival Project GW, an M & E specialist, took part in the development of study tools EM edited the manuscript PW supervised field data collection.

## Annex IV: CHW Profile Grid

**District ..............Location..............Sublocation..............**

**Table:**

CHW Code	Age	Sex	Marital status	Highest level of Education	Selected by	Period served	Household coverage	Supervisor	Supporter
									
									
									
									
									
									
									
									
									
									
									
									
									
									
									
									

**Annex V: CHW Knowledge Assessment Tool**

Name: ____________________________________________

1. Name four activities of a Community Health Worker in community based mother and newborn care.

____________________________________________________

____________________________________________________

____________________________________________________

____________________________________________________

2. During pregnancy, name 3 essential care interventions that a pregnant woman should receive from ANC.

____________________________________________________

____________________________________________________

____________________________________________________

3. What are three danger signs for a pregnant woman during pregnancy?

____________________________________________________

____________________________________________________

____________________________________________________

4. Explain three ways the CHW promotes a safe birth.

____________________________________________________

____________________________________________________

____________________________________________________

5. Name three danger signs that can appear in the baby or mother during birth and in the minutes after delivery.

____________________________________________________

____________________________________________________

____________________________________________________

6. What is kangaroo mother care and for whom especially is it used? What? For Whom?

____________________________________________________

____________________________________________________

____________________________________________________

7. Name three essential care interventions in the home to protect the newborn baby?

____________________________________________________

____________________________________________________

____________________________________________________

8. Name three danger signs that can appear in newborns?

____________________________________________________

____________________________________________________

____________________________________________________

9. Name three danger signs that can appear in a woman after giving birth?

____________________________________________________

____________________________________________________

____________________________________________________

Explain what you would do if a 3 day old newborn was very sleepy and difficult to wake up? What could be the problem?

_____________________________________________________

**Annex VI: CHW Case Observation Checklist** (allow use of all job aids)

**Table:**

Date of visit:		
Researcher:	**YES (1)**	**NO (0)**
**Records and supplies**		
Has list of pregnant women in area of coverage		
For each pregnant women, schedules 2 visits during pregnancy noted on List of Pregnant Women or calendar		
Has list of mothers having newborns		
Schedules post natal home visits		
Has all job aids (counseling cards, registers, equipment)		
**TOTAL score** (out of 5)		
**Good communication skills**		
Greeting.		
Explains why she is visiting today.		
Acts with confidence.		
Speaks in a gentle tone of voice, shows respect		
Uses simple words in local language.		
Uses any training aids appropriately		
Asks the woman if s/he has any questions.		
Answers clearly.		
Thanks her for visit.		
Says when s/he will return.		
**TOTAL score** (out of 10)		
**Hand Washing**		
Removes bracelet and watch		
Wets hands and arms to elbow		
Applies soap and scrub arms, hands and nails		
Rinses with clean water		
Air dries with hands up		
**TOTAL score** (out of 5)		
**Pregnancy Home Visit 1**		
Good Communication Skills(Above)		
Uses counseling cards/referral notes		
Counsels on importance of ANC		
Counsels on danger signs during pregnancy		
Counsels on health facility delivery		
Counsels on good nutrition and rest		
Counsels on individual birth plans		
Completes Pregnant Mothers register		
**TOTAL score** (out of 8)		
**Pregnancy Home Visit 2**		
Good Communication skills (above)		
Uses counseling cards appropriately		
Counsels on Birth Plan		
Counsels/screens for danger signs		
Counsels on breastfeeding		
Counsels on good nutrition and rest		
Counsels on immediate newborn care		
Completes Mother and Newborn Form correctly		
**TOTAL score** (out of 8)		
**Postnatal home visit**		
Good Communication skills (above)		
Uses counseling cards appropriately		
Conducts immediate newborn care (drying and wrapping in warm clothes)		
Assists in initiation of breastfeeding within one hour)		
Provides cord care		
Observes a breastfeed		
Teaches correct positioning and attachment appropriately		
Assesses newborn (weight, respiration, temperature)		
Provides eye care		
Counsels on continued newborn care		
**TOTAL score** (out of 10)		
**Skills checklist**		
Appropriately warms the newborn		
Correctly weighs the newborn		
Correctly counts respiratory rate		
Correctly takes newborn temperature		
**TOTAL score** (out of 4)		

Scores for each parameter will be ranked on an ordinal scale as below average, average and above average performance

**Annex VII: Client Interview Tool-Consultation Quality Index Tool (CQI-2 Tool)**

A: Consultation process measure- CARE (Consultant and Relational Empathy)

Please ask the client and rate the following statements about this CHW home visit consultation

Please tick one box for each statement and answer every statement.

How was the CHW at … Poor Fair Good V Good Excellent

1. Making you feel at ease … ◻ ◻ ◻ ◻ ◻

(being friendly and warm towards you, treating you with respect; not cold or abrupt)

2. Letting you tell your ‘story’ … ◻ ◻ ◻ ◻ ◻

(giving you time to fully express yourself in your own words; not interrupting or diverting you)

3. Really listening … ◻ ◻ ◻ ◻ ◻

(paying close attention to what you were sayings; not Looking/ facing away as you were talking)

4. Being interested in you as a whole person … ◻ ◻ ◻ ◻ ◻

(asking/knowing relevant details about your life, your situation; not treating you passively)

5. Fully understanding your concerns … ◻ ◻ ◻ ◻ ◻

(communicating that s/he had accurately understood your concerns; not overlooking or dismissing anything)

6. Showing care and compassion … ◻ ◻ ◻ ◻ ◻

(seeming genuinely concerned, not being indifferent or ‘detached’)

7. Being positive … ◻ ◻ ◻ ◻ ◻

(having a positive approach and a positive attitude; not negative about your problems/concerns)

8. Explaining things clearly … ◻ ◻ ◻ ◻ ◻

(fully answering your questions, explaining clearly, giving you adequate information; not being vague)

9. Helping you to take control … ◻ ◻ ◻ ◻ ◻

(exploring with you what you can do to improve your own or child’s health; encouraging rather than ‘lecturing’ you)

10. Making a plan of action with you … ◻ ◻ ◻ ◻ ◻

(discussing the options, involving you in decisions as much as you want to be involved; not ignoring your views)

B: Consultation outcome measure (Patient/client enablement Instrument-PEI)

Please complete these other questions about the home visit consultation by the CHW

As a result this visit by the CHW, do you understand/feel… ◻ ◻ ◻ ◻ ◻ (please tick one box in each row)

Much better Better Same or less Not applicable

Understand pregnancy/newborn care ◻ ◻ ◻ ◻ ◻

Identify danger signs in pregnancy/newborn ◻ ◻ ◻ ◻ ◻

Can take appropriate action ◻ ◻ ◻ ◻ ◻

C: Continuity measure

Very well Well Somehow Don’t know

1. How well do you know this CHW? ◻ ◻ ◻ ◻ ◻

Very good Good Somewhat good No comment

2. What do you say this CHW is ◻ ◻ ◻ ◻ ◻
